# Editorial: Prognosis prediction and risk stratification in head and neck cancer

**DOI:** 10.3389/fonc.2022.1037001

**Published:** 2022-12-30

**Authors:** Tengxiang Li, Jie Lu, Yong Yin, Qingtao Qiu

**Affiliations:** ^1^ Department of Radiation Oncology Physics, Shandong Cancer Hospital and Institute, Shandong First Medical University and Shandong Academy of Medical Sciences, Jinan, China; ^2^ School of Nuclear Science and Technology, University of South China, Hengyang, China

**Keywords:** head and neck cancer, prognosis, molecular biomarker, imaging biomarker, machine learning

Head and neck cancer is the seventh most common cancer worldwide, accounting for 3% of all cancers. For patients with early-stage disease, either surgery alone or definitive radiation therapy alone results in an excellent treatment outcome. However, the majority of newly diagnosed patients present with local-regionally advanced disease and require multimodality treatment. The treatment outcomes of these patients are far from satisfactory. It is widely recognized that treatment outcome is determined by a complex interaction of multiple factors, including biological, clinical, treatment, and environmental factors. However, current treatment decisions primarily rely on the clinical stage, which does not always reflect the variable clinical course and long-term outcomes. Recent advances in radiomics, genomics, proteomics, microbiome, AI technique and machine learning, and other potential imaging, biological, and clinical factors, alone or in combination, may provide new insights into the prognosis prediction and treatment response in head and neck cancer. Risk stratification based on these prognostic and predictive biomarkers will help design future clinical trials and personalized treatment strategies in head and neck cancer.

This paper aims to present the latest advances in basic, translational, and clinical research findings on predictions of treatment outcome and response and risk stratification in head and neck cancer, including nasopharyngeal carcinoma, and how these affect future clinical trials and treatment decisions in head and neck cancer.

## 1 Head and neck squamous cell carcinoma

By analyzing gene expression patterns in The Cancer Genome Atlas (TCGA) head and neck squamous cell carcinoma (HNSCC) dataset and GSE37991 dataset, Mai et al. examined possible prognostic indicators in head and neck carcinoma. The common differentially expressed metabolic enzymes were used to identify six expressed metabolic enzymes (DEMEs). When investigated on a modest scale, this metabolic enzyme-based risk signature proved to be more accurate at predicting the prognosis of HNSCC than tumor, node, and distant metastasis (TNM) stage since it was simplified to six genes. While taking into account the loss of specific clinicopathological information, clinical data collection techniques and management procedures must be strictly adhered to for the application of this gene signature in real-world situations.

Deoxyribonucleic acid (DNA) repair capacity and the tolerance of DNA to radiation damage varies in cancers and healthy tissues, and they are linked to clinical manifestations, including cancer resistance and side effects. Ming’s et al. developed a 13-diagnostic-related groups (13-DRG) signature for the prognosis of HNSCC, which could reliably and independently predict the patient’s clinical outcome. The researchers also revealed the immune landscape, tumor mutation burden, and sensitivity to chemotherapy drugs in various risk groups, all of which may be used to inform clinical treatment choices.

The prospective evaluation of patient characteristics and the identification of novel potential prognostic biomarkers for cutting-edge treatment options is a crucial topic for stratifying personalized treatment for recurrent/metastatic HNSCC (RM HNSCC). The implementation of a relatively detailed assessment technique will make it easier to identify individuals who will likely benefit from immunotherapy, salvage surgery or (re)irradiation. In the first line of treatment for RM HNSCC, a variety of variables affect treatment decisions as indicated in a questionnaire by Klinghammer et al., including performance status, programmed cell death ligand 1 (PD-L1) expression, time from last systemic treatment above or below 6 months, and disease burden.

To evaluate the prognostic performance of HNSCC, including overall survival and immune profile performance, Gao et al. created a lipid-related prognostic signature. Their study suggests that predictors created for aberrant lipid metabolism have the potential to be used in the therapeutic assessment of HNSCC.

Individualized precision medicine and the assessment of cancer prognosis have become attractive study areas. To objectively quantify and identify trustworthy and clinically useful biomarkers, Moratin et al.’s research performed immunohistochemical staining and digital pathology algorithms. Overall and progression-free survival estimates were made with a substantial degree of accuracy using a combined score combining the epidermal growth factor receptor (EGFR) expression, neck node status, and immune cell characteristics.

For immune checkpoint inhibitors (ICI) therapy response, different immune cell distribution patterns within tumors may be essential. HNSCC patients have a relatively moderate response rate to therapy due to the absence of prognostic indicators. According to Idel et al.’s analysis of the spatial distribution differences between each immune cell type, programmed death-1 (PD-1) and PD-L1 expression levels in certain tumor types may be important for predicting treatment response.

Exome sequencing was undertaken by Desai et al., and this analysis revealed that several tumor suppressor genes had mutations. If those genes were driver genes and had the potential to be druggable, they might be the subject of an actionable mutation.

A significant prognostic factor for HNSCC is the incidence of neck lymph node metastasis (LNM). As a result, understanding the molecular mechanisms involved in HNSCC LNM has important clinical ramifications for risk assessment. Zhang et al. used Gene Ontology enrichment analysis to create a risk score for identifying lymphocytes that infiltrate tumors. When used to predict the survival of patients with HNSCC, the prediction model demonstrated discrimination capacity and agreement.

In a meta-analysis of the literature on the role of microRibonucleic acid (RNA)s in the clinical manifestations of HNSCC treatment, Qiu et al. discovered that microRNAs are highly accurate at identifying recurrent, metastatic, and lymph node metastatic HNSCC. This finding suggests risk grading and individualized treatment of patients in the clinic for abnormal microRNA manifestations.

In bioinformatics research, the investigation of diverse microlevel prognostic indicators is a prominent topic. Shen et al. developed a long noncoding long non-coding (Lnc)RNA prognostic signature for HNSCC, which had a higher impact on risk assessment but requires more research to demonstrate its potential practical value.

A common regulator of messenger (m)RNA expression called N6-Methyladenosine has drawn growing research interest. Although the importance of m6A in several biological processes, including the development and spread of malignancies, has been well established, studies of its potential on the tumor immune microenvironment (TIME) are few and far between. According to Yang et al.’s findings, m6A regulators and the TIME have a strong relationship. This has implications for research into immunotherapy and chemotherapy regimens for HNSCC.

Patients with HNSCC still receive treatment based on their disease stage and tumor site rather than tumor biology. Numerous biomolecular markers have been proposed to identify primary and secondary cancers in the early stages of the disease, including proteins, DNA, RNA, and microRNAs. Immune checkpoint inhibitors have become a novel therapeutic option for HNSCC, which is an immunosuppressive disease. It would be beneficial to look at how immune cells and their regulators function in the tumor microenvironment of HNSCC. Because RNA-binding proteins (RBPs) are essential for the post-transcriptional control of genes, it is important to investigate how RBPs relate to HNSCC. An immune-related RBP predictive signature was developed by Ming et al.’s.

The majority of patients often receive a diagnosis of advanced HNSCC because of the asymptomatic nature of the early disease stages and the dearth of reliable screening techniques. Effective biomarkers must be found quickly in order to help doctors anticipate clinical outcomes with accuracy and provide references for specialized medical treatments to fight HNSCC. Nuclear transporter factor 2 (NTF2) was found as a potential diagnostic and prognostic biomarker for HNSCC *via* extensive analysis of its function by Xuan et al., which included RNA sequencing data and the associated clinical information.

Cisplatin is used as main or adjuvant (radio)chemotherapy for squamous cell carcinoma of the head and neck. There are two basic dosage regimens currently used, and the highest cumulative total dose of cisplatin is desired for the best results. The 3-weekly regimen had a larger cumulative total dose, according to Jungbauer et al.’s retrospective research. It can be concluded that the 3-weekly regimen is superior to the weekly regimen because this appears to favorably correlate with patient outcome. Functional organ systems, particularly those of the kidneys and bone marrow, are linked to a higher cumulative total dosage and can be thought of as predictive factors.

Primary surgery is followed with risk-adapted adjuvant radiotherapy (RT)/chemoradiotherapy (CRT) or definitive CRT for tumors that are functionally inoperable as the standard of care for locally advanced oral cavity cancer (LA-OCC). Patients with locally advanced HNSCC experience local recurrences and distant metastases after receiving combined modality therapy, and local control rates for the LA-OCC subgroup are still lower than those for LA-HNSCC, with the majority of locoregional failures occurring in the area of preceding RT. A prospective, single-arm experiment was initiated by Grün et al. to examine the feasibility and early efficacy of neoadjuvant chemoradiotherapy (nCRT) followed by surgery in LA-OCC, with a special emphasis on potential prognostic biomarkers.

The identification of cancers that will respond to treatment is required since targeting the immune system has proven to be a successful therapeutic approach for the management of different tumor types. Saiz-Ladera et al. discovered a collection of gene combinations connected to a greater presence of immune effector cells that are associated with better outcomes in HNSCC. This novel signature also recognizes a subset of cervical squamous cell carcinoma (CSCC), but not esophageal or lung squamous cell carcinoma (SCC). These findings can serve as a guide for choosing the focus of future studies.

It is important to determine whether human papillomavirus (HPV) immunotherapy effectiveness has a potential relationship with the tumor immune microenvironment since HPV+ or HPV- HNSCC patients have distinct prognostic outcomes.Wu et al.’s study used a single-cell RNA sequencing dataset and evaluated CD8+ T-cell based genes including ACAP1 (adenosine diphosphate ribosylation factor GTPase-activating proteins with Coiled-coil, Ankryin repeat and PH domains 1), ankyrin repeat domain 28 (ANKRD28), chromosome 12 open reading frame 75 (C12orf75), and mannose-6-phosphate receptor (M6PR) that could predict prognosis and immunization-correlated treatment responses.

## 2 Nasopharyngeal carcinoma

Nasopharyngeal carcinoma (NPC), which makes up a sizable fraction of head and neck tumors, has a considerable regional incidence. In a literature meta-analysis carried out by Chiang et al. for the eighth edition of TNM staging, clinical indicators were screened to suggest prognosis, such as upstaging paranasal sinus to T4. The conclusions obtained from this meta-analysis were all common clinical indicators, which are convenient and simple to use.

The commonly used clinical TNM staging may need to be taken into account for factors like varied EGFR expression for different outcomes over the same period since it does not specifically predict each patient’s prognosis in nasopharyngeal carcinoma. High EGFR expression was found to be strongly related with poor overall survival (OS) and disease-free survival (DFS) in Chen et al.’s meta-analysis. It should be emphasized in practice that there was no significant link between various EGFR expression and progression-free survival (PFS), distant metastasis-free survival (DMFS), OS, etc., in the subgroup analysis.

Nasopharyngeal carcinoma is usually diagnosed beyond stage I because of the insidious location of the primary region. Induction chemotherapy combined with concurrent radiotherapy is commonly used in the clinical treatment of patients with locally advanced nasopharyngeal carcinoma. In Xiong et al.’s study, a comparison of the effectiveness of several chemotherapy regimens demonstrated that TPF (taxanes, cisplatin, and 5-fluorouracil) and TP (taxanes and cisplatin) led to different outcomes due to differences in toxicities in patients with NPC at N2-3 stages.

New options and approaches for investigating the diagnosis, therapy, and prognosis of NPC have been presented by the combination of radiomics and multimodal imaging. To diagnose and treat NPC, radiomics and machine learning have been combined. However, model selection is where machine learning in radiomics is most commonly used. Radiomics, a technology for extracting information from depth images, can help with NPC diagnosis and treatment, but it also presents a number of difficulties, including the need for large datasets for the development of tumor models, data sharing between various medical institutions, and different imaging protocols, as summarized by Zhang et al. To integrate radiomics models into clinical practice, significant progress is still needed. For radiomics to promote individualized and intelligent treatment, more forward-looking research and applications are needed.

In a different meta-analysis on nasopharyngeal carcinoma, author Jing et al. analyzed the clinical manifestation of patients with various blood types and discovered that the incidence of nasopharyngeal carcinoma was lower in the Chinese population with blood group O. Worse 5-year OS, locoregional relapse-free survival (LRRFS) or DMFS rates were discovered in patients with blood group O. The study did not, however, analyze the distribution of blood types across various geographies or look into whether a person’s blood type may be related to their onset of a certain disease.

The main factors influencing the occurrence of hypothyroidism in nasopharyngeal carcinoma patients following radiotherapy, specifically after intensity-modulated radiotherapy (IMRT), are the thyroid gland’s volume and dosage. According to Shen et al.’s normal tissue complication probability (NTCP) model, which was built using multivariate construction, the best strategy to safeguard thyroid function was to reduce the average dose in the thyroid as much as possible.

Radiation therapy, along with other treatments including chemotherapy, is generally used to treat NPC. To improve the effectiveness of radiation therapy and limit the toxicity to normal tissue, the tumor target area of NPC ought to be precisely defined. According to Yan et al.’s study, the 50% standard uptake value (SUV)max threshold regimen for gross tumor volume (GTV) delineation with dose-painting appeared to be superior to the visual criterion or SUV2.5 threshold when it related to defining tumor volume in locoregionally progressed NPC with no increased toxicity.

Emerging radiomics has made it possible to reveal hidden biological characteristics and the genetic relationship between tumor and organ structures. There is growing evidence in the literature that radiomics may accurately predict treatment response based on volume shrinking in a variety of cancer types. To determine if patients with NPC were eligible for adaptive radiotherapy (ART), Lam et al. looked at the function of several multiorgan omics-based prediction models. Given the rising demand for ART in this particularly sensitive population of cancer patients during the period of IMRT, this study may offer the community helpful insights toward creating ART screening tools in the future.

According to the guidelines for the treatment of NPC, except for T1N0M0, which is treated with radiation alone, T2N0M0 and T1/2N1M0, and other stages are usually treated with a combination of radiotherapy and chemotherapy. Li et al. established residual volume of lymph nodes during chemoradiotherapy, which is useful in estimating 4-year OS, PFS and DMFS in NPC patients.

Peng et al. evaluated the therapeutic effects of CRT preceded by induction chemotherapy, which could consist of docetaxel plus cisplatin (TP), TP plus 5-fluorouracil (TPF) or cisplatin plus 5-fluorouracil (PF). These long-term follow-up studies are essential, and the results on OS and toxicity can be utilized when selecting chemotherapy regimens for patients with locally advanced NPC.

In addition to being extremely accurate in identifying soft tissues, magnetic resonance imaging (MRI) can distinguish between tumor invasion of bone structures and other symptoms in locally progressed NPC. Kang et al.’s study developed radiomics-based models of MRI images, which used pre- and in-treatment pictures with greater continuity and had superior local recurrence free survival (LRFS), DMFS, and OS predictions. These models were better at predicting disease progression or death. This concept may potentially be used in the diagnosis of various illnesses and as a tool for evaluating treatment effectiveness and prognosis, among other uses for MRI.

According to Liang et al.’s study, the eighth edition of the American Joint Committee on Cancer (AJCC) staging system for NPC in an endemic area integrating into the pretreatment neutrophil-to-lymphocyte ratio (NLR) may improve the ability to separate and discriminate between N classifications, but not within T classifications. Furthermore, the addition of adjuvant chemotherapy to concurrent chemoradiotherapy may be beneficial for individuals in the recursive partitioning analysis (RPA) 4 group. This statistical work aids in guiding the selection of a clinical treatment regimen.

The ability of epstein-barr virus (EBV) DNA levels in plasma before and after various treatments to predict the prognosis of NPC was examined in Zhu et al.’s study. It was discovered that pre-neoadjuvant chemotherapy (pre-NACT) and post-NACT EBV DNA levels can predict survival outcomes like PFS and OS in patients with NPC. The inability to forecast radiation efficiency using EBV DNA continues to be a significant drawback in its use.

Effective therapeutic target sites and corresponding treatment may improve survival of NPC patients. Li et al. explored the mechanism of action in NPC species based on the enrichment of MiR-483-5p microRNA in plasma, biopsy tissue, and tumor cells of patients with NPC in prior studies. This targeting site exists in tumor cells and has a high potential value in targeted immunotherapy.

Fewer studies have been conducted on distal lymph node metastasis in individuals with recurrence, compared to more studies on cervical lymph node metastasis in NPC. Subphrenic lymph node metastasis predicts a worse prognosis, according to Zhang et al.’s study, which was a two-center, small-sample study. The results of the study have strong implications for early recurrence detection and accurate prognosis assessment after expanding the geographical area, centers, and samples.

KIF15, a member of kinesin-12 family, has been shown to have an impact on the occurrence and progression of some types of human cancer and is essential for numerous biological processes. However, there have not been many thorough analyses on the function of KIF15 in human malignancies, and it is still unknown how KIF15 affects NPC diagnosis and prognosis. KIF15 was discovered to be highly expressed in NPC tissues, and this was associated with a bad prognosis for NPC. KIF15 might be used as a therapeutic target in the management of NPC. Mi et al. examined KIF15’s diagnostic and prognostic potential in NPC through a pancancer investigation.

## 3 Oral squamous cell carcinoma

Although many therapeutic approaches for oral squamous cell carcinoma (OSCC) have demonstrated encouraging results in the treatment of OSCC in recent years, the 5-year survival rate is still low. Ding et al. discovered preoperative the neutrophil-to-lymphocyte ratio (NLR), lymphocyte-to-monocyte ratio (LMR), neutrophil-to-white blood cell ratio (NWR), and lymphocyte-to-white blood cell ratio (LWR) in the peripheral blood as prognostic predictors of OSCC using studies like Kaplan−Meier curves, which are helpful in predicting OSCC progression.

OSCC patients with HPV-negative status typically have poor clinical outcomes and worse treatment outcomes. In patients with HPV negative OSCC who were receiving radiotherapy, Ai et al. discovered that CD68+ macrophage infiltration was related to poor overall survival. Radiation therapy, Poly(I:C), and drugs that target HMGB1 may improve OSCC’s prognosis and responsiveness.

Perineural invasion (PNI), a crucial aspect of tumor invasion from a histological standpoint, aids in the spread of the tumor, however the prognostic significance of PNI is still up for debate. Traditional PNI was subclassified by Fu et al. to worst pattern of PNI (WPNI), and WPNI 3 was able to predict patients’ prognoses on its own. Trichotomy provided more careful and exact pathology evidence for tumor-nerve interactions in OSCC patients.

Radiotherapy, chemotherapy, and other treatments are ineffective against hypoxic tumors. The recognition of various hypoxia patterns and the creation of a hypoxia-related risk score may improve our understanding of the tumor microenvironment of OSCC, according to Li et al.’s study. Determining the hypoxic state of tumors in various patients is a prerequisite for targeted and precise patient treatment.

In individuals with OSCC, the prognosis and immunotherapy response rates are dismal. The fundamental processes for how the tumor microenvironment affects the prognosis and development of tumors are still unknown. According to Zhu et al.’s research, the immune-related gene signature can predict overall survival and help OSCC patients receive individualized care. It can also identify patients who might benefit from immunotherapy as well as treatments that concentrate on metabolic pathways, DNA damage or repair, and spliceosomes.

Of all malignancies of the oral cavity, squamous cell carcinoma is the most prevalent. The prognosis is influenced by many variables, including T stage on size and depth of invasion, and degradation of the mandibular bone. In patients with gingivo-buccal complex squamous cell carcinoma (GBC-SCC), Mahajan et al. studied the pattern of mandibular involvement and its impact on oncologic outcomes. He proposed a staging system based on the pattern of bone involvement (MMC: Marrow and mandibular canal staging system), only marrow with or without mandibular canal involvement is linked to worse survival outcomes.

## 4 Papillary thyroid carcinoma

The incidence of papillary thyroid carcinoma (PTC), one of the most frequent malignant carcinomas of the endocrine system, is rising globally. Although there are now diagnostic and therapeutic options for thyroid cancer, the prognosis is still unknown. Cancer invasion, malignancy, metastasis, and medication resistance are all impacted by autophagy. Long noncoding RNAs (lncRNAs) have been implicated in the development of several forms of cancer, according to recent study. However, it is still unclear how the autophagy process and lncRNAs are linked, as well as the relevance of autophagy-related lncRNA for risk assessment, medication sensitivity prediction, and prognosis prediction in PTC patients. Based on the expression patterns of lncRNAs associated with autophagy, Mu et al. developed a unique risk classification system for PTC that may be utilized for prognosis prediction, drug sensitivity prediction, and risk assessment.

PTC is regarded as a benign, slow-growing tumor with a favorable prognosis and minimal malignancy; nonetheless, some individuals still have early cervical lymph node metastasis (CLNM), which increases the chance of local recurrence. The typical symptom of CLNM in PTC is a lateral cervical lymph node metastasis from the central lymph node. Although some PTCs may not develop central lymph node metastasis, they may develop direct lateral lymph node metastasis (LLNM). The most crucial factor for deciding the surgical technique prior to surgery is CLNM, which is the biggest risk factor for local recurrence and the prognosis of PTC patients. Hu et al. developed a nomogram that demonstrated an excellent prediction of CLNM in patients with PTC and was simple to employ.

PTC, which makes up a large portion of the histological subtypes of thyroid cancer, has a fast-rising morbidity and mortality rate due to lymph node metastases or distant metastases. We must develop a deeper understanding of the etiology of PTC patients with distant or lymph node metastases. Asporin was used by Zhan et al. to identify PTC patients with or without lymph node metastases using a TMT-based quantitative proteomics technique. Asporin’s high expression in PTC tumorous tissues is a risk factor for a poor prognosis.

Alternative splicing (AS) events from Liu et al.’s study, through a limited analysis, could be regarded as trustworthy prognostic biomarkers for PTC. AS is crucial for the diversity of proteins and is closely linked to tumorigenicity, and these modifications are crucial for biological processes.

## 5 Oral cavity squamous cell carcinoma

Patients with distant metastases (DM) from oral cavity squamous cell carcinoma (OCSCC) have poor prognoses, and there are few reliable models for DM prediction. Although the DM growth mechanisms of the lymphatic and blood vessel systems may be different, DM development can happen directly through either of these systems. To build models for predicting DM in three years, Lu et al. used grouping factors and individually tailored micro parameters, such as age, surgical margin, early locoregional recurrence, lymphocyte-to-monocyte ratio, and presence of lymphovascular invasion.

The Cancer Genome Atlas’ oral cavity malignancies have a unique clinicopathological characteristic called partial epithelial-mesenchymal transition (p-EMT). The tumor stroma must provide extra assistance to the p-EMT cells, which are at the invasion front, in order for them to move in concert. This assistance includes track clearance, extracellular matrix remodeling, and immune evasion. By combining disease-matched xenograft tissue and single-cell RNA-seq findings, Liu et al. found that transforming growth factor beta induced (TGFBI) and hyaluronidase genes 1 (HYAL1) could act as reliable predictive biomarkers for the prevention of oral cancer.

## 6 Parotid carcinoma

Multiple factors have been linked to the prognosis of patients with parotid carcinoma (PC). A competing risk nomogram developed in Li et al.’s single-center, long-term research can be utilized to estimate cancer-specific mortality in PC patients. For use as a guide for evaluation in the clinic, this nomogram must be verified across a number of locations.

Accurate computerized dose prediction can considerably increase the effectiveness and safety of clinical planning. In contrast to typical automatic plans, which concentrate on conventional accelerators, the Liu et al.’s study looked at tomotherapy plans using a patient-specific gap between organs at risk (OARs) and planning target volumes (PTVs) in the model-building process to improve a method for creating automatic tomotherapy planning.

## 7 Oropharyngeal squamous cell carcinoma

By examining imaging characteristics, radiomics is utilized to determine whether oropharyngeal squamous cell carcinoma (OPSCC) is caused by HPV+ or HPV- and to determine the prognosis. Song et al.’s, which used radiomics for the risk assessment of patients with OPSCC to enable individualized therapy and enhance outcomes, provided evidence for the role of radiomics in this regard.

## 8 Hypopharyngeal squamous cell carcinoma

The prognosis of patients with hypopharyngeal squamous-cell carcinoma (HSCC), a head and neck cancer, varies greatly. According to research by Tian et al., various demographic traits, clinicopathological variables, and treatment modalities are highly connected with the survival results of HSCC patients. The data are simple to gather, demonstrating the simplicity of using this nomogram in clinical practice to support the clinical evaluation of the risk level of HSCC patients and the creation of tailored treatment plans.

## 9 Differentiated thyroid cancer

For individuals with low-risk differentiated thyroid carcinoma, total thyroidectomy (TT) or lobectomy without radioactive iodine (RAI) is increasingly the standard of care (DTC). It is important to pay attention to the techniques used to evaluate the effectiveness of the therapies and the suggestions that might be made, especially in light of the numerous side effects. According to Dong et al.’s study, there is no regular advice for RAI following surgery in low-risk DTC patients due to patterns of suppressed serum thyroglobulin (Tg) and anti-thyroglobulin antibody (TgAb) levels and neck ultrasonography results.

## 10 Salivary gland carcinoma

PD-L1 expression and prognostic significance in high-grade salivary gland carcinoma (SGC) is one of the predictors of immunotherapy efficacy. According to Fang et al.’s research, PD-L1 expression in tumor cells of high-grade SGCs rather than in immune cells was a marker of a poor prognosis and was strongly correlated with tumor stage. This finding may indicate that treatment should focus on patients with this type of protein expression.

## 11 Maxillary sinus carcinoma

Maxillary sinus carcinoma (MSC) makes up a small percentage of head and neck cancers; studies based on just one medical facility have small sample numbers. MSC has a concealed anatomical site and a complicated neighboring connection that results in a vague prognosis. In people with MSC, Hu et al.’s competing risk nomogram was successful in calculating the risk of cancer-specific death (CSD).

## 12 Salivary duct carcinoma

Salivary duct carcinoma (SDC) is a rare, extremely aggressive tumor that can develop both spontaneously and as part of pleomorphic adenoma. A majority of SDCs express the androgen receptor (AR), and approximately 40% are human epidermal growth factor receptor 2 (HER2)-positive. Treatments targeting AR and HER2 have recently been developed as a potential optional therapy in recurrent/metastatic or unresectable locally advanced SDCs based on these biomarker findings. For patients with SDC who tested positive for AR, AR-targeted treatment showed comparable effectiveness and less toxicity than traditional chemotherapy. Additionally, HER2-targeted therapy outperformed conventional or AR-targeted therapy in terms of effectiveness, with a greater response rate in HER2-positive SDC patients. However, choosing the best course of action is still challenging since SDCs frequently express both HER2 and AR. High expression of enhancer of zeste homolog 2 (EZH2) and histone H3 lysine 27 trimethylation (H3K27me3) in SDC was shown to be a potential indicator of the ineffectiveness of AR-targeted treatment, according to Saigusa et al.’s research.

## 13 Sinonasal carcinomas

Sinonasal carcinomas (SNCs) are difficult to categorize. As a result, prognosis and response prediction to nonsurgical treatment are frequently incorrect. The lack of prognostic and predictive tools is an unmet need, and the clinicopathological characteristics of the disease are the first logical source of information to be examined. In comparison to the current World Health Organization (WHO) categorization, Ferrari et al.’s analysis of cytomorphological, histomorphological, and locoregional extension offered a more accurate prediction. SNC chemo-radiosensitivity prediction, however, was not achieved.

## 14 Malignant myoepithelioma of the head and neck

The features and survival rates of malignant myoepithelioma of the head and neck (HNMM), a rare tumor, are not well defined. It will be important to investigate the epidemiology of HNMM and determine the criteria that will affect the prognosis of the condition. According to Wang et al., patients with HNMM frequently have a good prognosis, and factors including distant metastasis, pathological grade, and the use of surgery all help them survive. To assist doctors in the clinical care of this uncommon disease, the undifferentiated pathological grade and M1 in the M category were independent prognostic markers to predict OS and disease-specific survival (DSS) for HNMM patients.

We look forward to more researchers contributing more ideas, validations, reviews, etc., on head and neck cancer for prognosis and risk assessment, including but not limited to imaging biomarkers, molecular biomarkers including DNA, EBV-DNA, and HPV-DNA, microbiome, artificial intelligence technique and machine learning, and other potential biological or clinical factors, in combination with *in vitro*/*in vivo* validation. Additionally, we look forward to the prediction of treatment outcome and response and the latest development and validation of predictive models for head and neck cancer. Furthermore, we look forward to the outcomes of clinical trials based on prediction models established from above research and updated information about or preliminary results of ongoing clinical trials.

## Author contributions

TL, JL, QQ and YY designed the study and wrote the manuscript. TL and QQ participated in the study designing and data collection. QQ and YY participated in offered guidance. All authors contributed to the article and approved the submitted version.

